# Standardized single-incision plus one-port laparoscopic left lateral sectionectomy: a safe alternative to the conventional procedure

**DOI:** 10.1007/s00423-021-02340-4

**Published:** 2021-12-06

**Authors:** Hirokatsu Katagiri, Hiroyuki Nitta, Takeshi Takahara, Yasushi Hasegawa, Syoji Kanno, Akira Umemura, Daiki Takeda, Kenji Makabe, Koji Kikuchi, Taku Kimura, Shingo Yanari, Akira Sasaki

**Affiliations:** 1 Department of Surgery, Iwate Medical University School of Medicine, 2-1-1 Idai-dori, Yahaba, Iwate 028-3609 Japan; 2 Department of Surgery, Fujita-Gakuen Health University School of Medicine, Aichi, Japan; 3 Department of Surgery, Keio University School of Medicine, Tokyo, Japan

**Keywords:** Laparoscopic, Hepatectomy, Liver resection, Reduced port, Left lateral sectionectomy, Training

## Abstract

**Purpose:**

Laparoscopic left lateral sectionectomy (LLLS) is a feasible and safe procedure with a relatively smooth learning curve. However, single-incision LLLS requires extensive surgical experience and advanced techniques. The aim of this study is to report the standardized single-incision plus one-port LLLS (reduced port LLLS, RPLLLS) technique and evaluate its safety, feasibility, and effectiveness for junior surgeons.

**Methods:**

Between January 2008 and November 2020, the clinical records of 49 patients who underwent LLLS, divided into the conventional LLLS (*n* = 37) and the RPLLLS group (*n* = 12), were retrospectively reviewed. The patient characteristics, pathologic results, and operative outcomes were evaluated.

**Results:**

A history of previous abdominal surgery in the RPLLLS group was significantly high (56.8% vs. 91.7%, *p* = 0.552). Notably, junior surgeons performed 62.2% of the conventional LLLSs and 58.4% of the standardized RPLLLSs. There were no significant differences between the two groups in terms of median operative time (121.0 vs. 113.5, *p* = 0.387), median blood loss (13.0 vs. 8.5, *p* = 0.518), median length of hospital stays (7.0 vs. 7.0, *p* = 0.408), and morbidity rate (2.7% vs. 0%, *p* = 0.565), respectively.

**Conclusion:**

This standardized RPLLLS is a feasible and safe alternative to conventional LLLS and may become the ideal training procedure for both junior surgeons and surgeons aiming to learn more complex procedures.

Laparoscopic liver resection (LLR) is a safe and feasible alternative to open hepatectomy for the treatment of liver disease. Previous studies have demonstrated that LLR results in less blood loss, less postoperative pain, shorter hospital stays, and lower morbidity compared with open resection [1, 2]. Moreover, the oncological outcomes of LLR are comparable with those of open liver resection [3]. The first account of laparoscopic left lateral sectionectomy (LLLS) was reported in 1996 [4, 5]. LLS 2 and 3 have since been established as equivalent or even better options compared with the same intervention performed by laparotomy [6].

Reduced port surgery has been drawing attention in the field of minimally invasive surgery. In particular, the progress on laparoscopic techniques and the various devices involved, including multi-access ports with multiple channels, has enabled the execution of single-port laparoscopic surgery (SPLS) [7]. Previous studies on SPLS indicate that it has advantages over the conventional multiport laparoscopic surgery, such as reduced length of hospital stay or better cosmetic outcomes [7–9]. In the LLR field, several studies on single-port laparoscopic liver resection have reported that it is feasible and as safe as traditional laparoscopic surgery for tumors in the left liver lobe [10–13].

In spite of the technical feasibility, safety, and clinical benefits of LLR, it is technically difficult because experience in both hepatobiliary and laparoscopic techniques is required and it does not have a steep learning curve [14–16]. A comparative analysis has found that LLLS is a feasible, safe, and efficient procedure that is associated with a quick, smooth learning curve [17–19]. We consider a standardized single-port plus one-port LLLS (reduced port LLLS, RPLLLS) to be a safe and feasible procedure for junior surgeons who are in the process of learning laparoscopic hepatectomy. The aim of the present study is to report the standardized RPLLLS technique and compare its surgical outcomes with those of conventional LLLS.

## Patients and methods

LLR has been performed at our institution since May 1997. From then until November 2020, a total of 792 patients consecutively underwent pure LLR. In 2003, pure LLLS was adopted, and 73 (9.2%) pure LLLSs were performed between January 2003 and November 2020. Beginning 2008, the conventional LLLS procedure has been standardized by trial and error. In 2013, the performance of the standardized RPLLLS was started. The indications for the standardized RPLLLS corresponded with those for conventional LLLS.

We conducted a retrospective study that enrolled 49 consecutive patients who underwent conventional LLLS and RPLLLS at Iwate Medical University between January 2008 and November 2020. This study protocol was approved by the institutional review board. All patients were informed about the procedure, and consent was obtained before surgery. Considering the learning time needed for surgeons to develop an adequate level of expertise in performing LLR, we only used the data from the 65 patients who underwent LLLS between January 2008 and November 2020. From this subset, we excluded 16 patients who underwent multiple hepatectomies during surgery, radiofrequency ablation concomitant to LLR, and donor hepatectomies.

The inclusion a were as follows: (1) patients who underwent LLLS, (2) patients with left lateral section lesions and (3) patients with no evidence of tumors in other parts of the liver or outside liver. The exclusion criteria were as follows: (1) patients with lesions spreading to other organs, (2) patients needing bile duct resection, (3) patients needing lymph node dissection, (4) patients who underwent multiple hepatectomies during surgery, (5) patients who underwent radiofrequency ablation concomitant to LLR, and (6) patients who underwent donor hepatectomy. The exclusion criteria for LLR were lesions spreading to other organs and patients requiring bile duct resection or lymph node dissection. These are indicated for laparotomy. Neither malignancy nor cirrhosis was exclusion criteria.

The subjects were divided into the conventional LLLS and RPLLLS groups according to the surgical treatment. Within the period of this review, 37 (75.5%) of the 49 LLLS patients underwent conventional LLLS, while 12 (24.4%) underwent the standardized RPLLLS. Two expert surgeons and seven junior surgeons performed LLLSs in this study. Since 2011, LLLSs have been performed by junior surgeons without considering patients’ background characteristics (e.g., cirrhosis, tumor location, and age). Since 2018, the standardized RPLLLSs have also been performed by junior surgeons without considering such patient characteristics. Each junior surgeon in this database performed approximately 10 open hepatectomies and 5–10 laparoscopic hepatectomies; they also assisted in 20–30 laparoscopic hepatectomies during their training.

The clinical records of the 49 patients were reviewed to extract the following information for analysis: patient characteristics (e.g., sex, age, American Society of Anesthesiologists physical status, body mass index, repeat hepatectomy, history of previous abdominal surgery, cirrhosis, and indocyanine green retention rates at 15 min), pathological results (e.g., diagnoses, largest tumor diameter, multiple tumors, surgical margin, and positive surgical margin), and operative outcomes (e.g., operated by junior surgeon, operative time, blood loss, conversion to laparotomy, length of hospital stay, morbidity, and mortality).

## Surgical technique

According to previous reports on the conventional LLLS procedure [19], it is characterized by the absence of the Pringle maneuver, traction of the round ligament with Endoloop™ (Ethicon Endosurgery; Cincinnati, OH, USA) ligature, minimal liver parenchymal transection without exposure of the portal pedicles or hepatic vein, and use of linear staplers to divide the portal pedicles or hepatic vein [20]. The required minimal liver transection is accomplished using a Harmonic Scalpel™ (Ethicon Endosurgery). The standardized RPLLLS is essentially similar to the conventional LLLS, except for the fact that it is characterized by the use of a flexible endoscope and the placement of an epigastric port. This port allows operators to access the left coronary and triangular ligaments easily, affords safe access to the edge of the left hepatic vein, assists in surgical operations without interfering with the endoscope during transection, and prepares for possible issues around the Glissonean pedicles or left hepatic vein dissection.

**Fig. 1:**
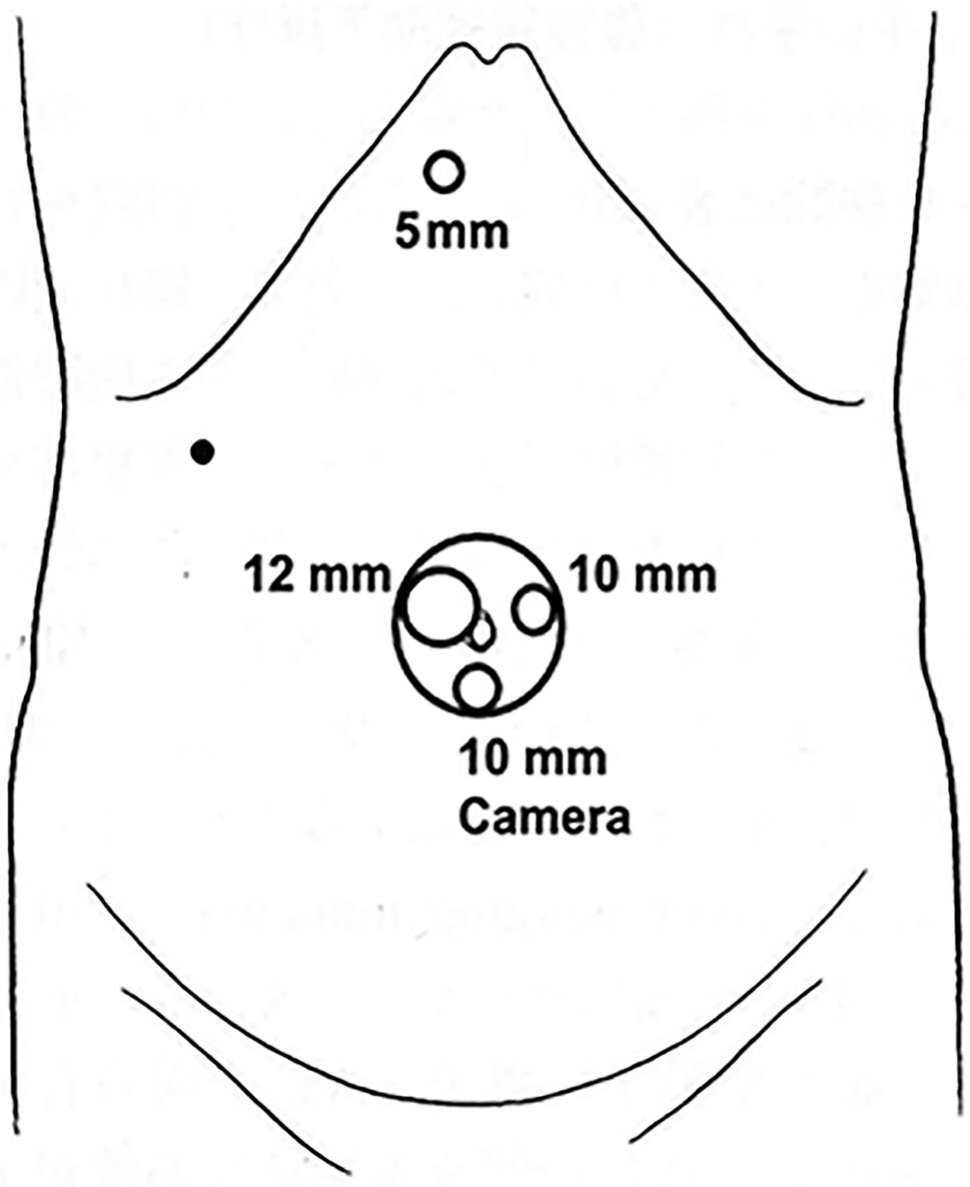
Trocar placement for the standardized RPLLLS. Filled circle is the point where the Endoloop is pulled out

**Fig. 2:**
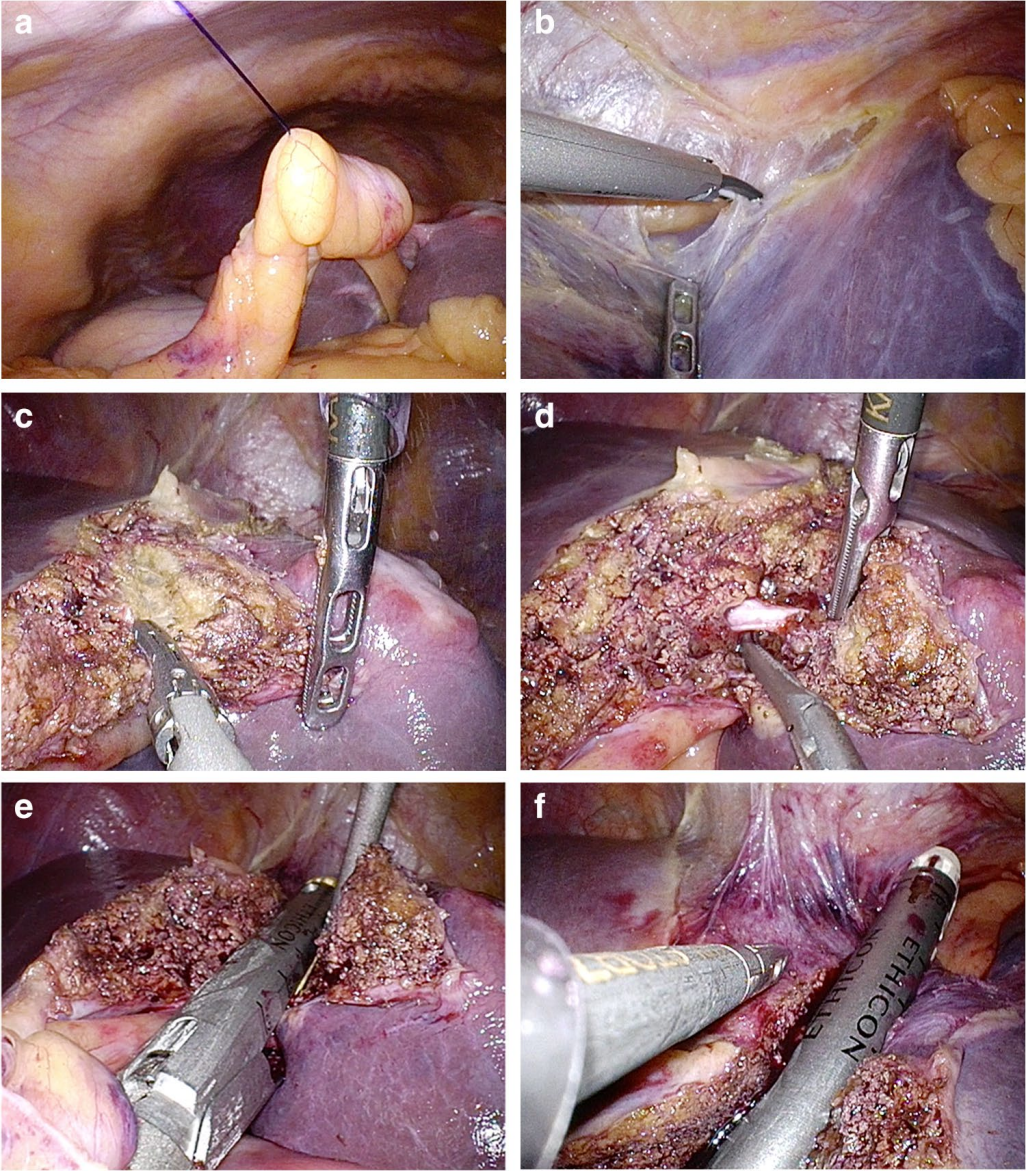
The liver traction with round ligament grasped by Endoloop (**A**), the left side hepatic ligament division through the epigastric port (**B**), the liver parenchyma transection to decrease the liver’s thickness (**C**), the portal pedicle division with the epigastric port assistance (**D**), and the portal pedicle (**E**) and left hepatic vein division using the linear stapler (**F**)

The standardized RPLLLS procedure is conducted by placing the supine patient in the reverse Trendelenburg position. The operator stands to the right of the patient with the assistant, while the scopist is on the patient’s left. In this procedure, all operations are performed using a GelPOINT™ (Applied Medical, Rancho Santa Margarita, USA) through a 4.0-cm umbilical incision. The GelPOINT is an access device with a large outer cap designed to increase the instrument distance. One 12-mm sleeve and two 10-mm sleeves are placed on the GelPoint, and one 5-mm trocar is placed at the epigastric site, as shown in Fig. 1. A carbon dioxide pneumoperitoneum is maintained at 8–12 mmHg. The standardized RPLLLS procedures are shown in Fig. 2A–F. The round and falciform ligaments are divided with a Harmonic Scalpel through the umbilical port. The round ligament is grasped with Endoloop ligatures and pulled to the right using an Endo Close™ (Medtronic; Dublin, Ireland) suturing device. The coronary and left triangular ligaments are divided through the epigastric port, and the surface of the hepatogastric ligament is divided with the Harmonic Scalpel to free the left lateral section. Following these preparatory steps, the liver is transected along a line just left of the falciform ligament using the Harmonic Scalpel or with the clamp–crush method without using the Pringle maneuver. The saline dripping monopolar and a soft-coagulation system are used for hemostasis. The liver parenchyma is transected to a depth of 1–2 cm with the Harmonic Scalpel to decrease the liver’s thickness and enable the insertion of an endoscopic linear stapler. Minimal transection is performed near the left hepatic vein. Note that neither the left hepatic vein nor the portal pedicles of segments II and III are exposed, but these vessels or pedicles may be divided if necessary. The operator assists these procedures with a suction tube or forceps through the epigastric or umbilical port. The assistant secures surgical field of view by applying tension to the liver transection surface through a 10-mm port on the assistant side at the umbilical site. Both portal pedicles, along with the surrounding liver tissue, are divided using a Powered Echelon Flex™ (Ethicon Endosurgery) stapler with a 60-mm gold cartridge. Thereafter, the left hepatic vein within the liver parenchyma is divided using the stapler with a 60-mm white cartridge. Bleeding is almost nonexistent after the division of the liver with these linear staplers. Finally, the specimen is extracted using a protective bag through an umbilical incision, and the pneumoperitoneum is decreased to 4 mmHg while hemostasis is confirmed. Prophylactic abdominal drains are not placed in any of the patients.

## Definitions

An expert surgeon was defined as a surgeon who had performed 50 or more laparoscopic liver surgeries as operator. A junior surgeon was defined as a surgeon who was learning to perform laparoscopic hepatectomy under the mentorship of expert surgeons. Surgical margin was defined as the diameter between the liver resection stump and the edge of a benign tumor or cyst. Postoperative morbidity was graded according to the Clavien–Dindo classification. Postoperative mortality was defined as any death occurring within 90 days of liver resection.

## Statistical analysis

The continuous variables are described as medians with interquartile range, whereas the categorical variables are described as totals and frequencies. Differences in groups were assessed through the Mann–Whitney *U* test for the continuous variables and the chi-square test or Fisher’s exact test (for expected counts of < 5) for the categorical variables. Statistical analysis was performed using the software JMP (version 13.2.0, SAS Institute, Cary, NC, USA). Variables with a *p* value < 0.05 were considered statistically significant.

## Results

The data of 49 patients, of whom 37 underwent conventional LLLS and 12 underwent standardized RPLLLS, were analyzed. The patients’ characteristics are shown in Table 1. All patients were classified as Child–Pugh class A (not shown). A history of previous abdominal surgery in the RPLLLS group was significantly high (56.8% vs. 91.7%, *p* = 0.552). Otherwise, except for the indocyanine green retention rates at 15 min, there were no significant differences in the patients’ age, sex, American Society of Anesthesiologists physical status, body mass index, repeat hepatectomy, and liver cirrhosis.

**Table 1:** Characteristics of patients undergoing LLLS

Characteristics	Conventional LLLS *n* = 37	RPLLLS *n* = 12	*p* value
Sex (male)	21 (56.8)	8 (66.7)	0.543
Age (years)	64.0 (26.0–86.0)	67.5 (52.0–81.0)	0.681
ASA-PS			0.379
1	9 (24.3)	1 (8.3)	
2	23 (62.2)	10 (83.4)	
3	5 (13.5)	1 (8.3)	
BMI (kg/m^2^)	22.2 (14.3–34.8)	21.2 (18.7–28.7)	0.756
Repeat hepatectomy	1 (2.7)	1 (8.3)	0.391
History of previous abdominal surgery	21 (56.8)	11 (91.7)	0.027
Cirrhosis	6 (16.2)	1 (8.3)	0.497
ICG 15R (%)	12.0 (5.0–36.0)	3.5 (1.0–14.0)	0.008

*ASA-PS* American Society of Anesthesiologists physical status, *BMI* body mass index, *ICG-R15* indocyanine green retention rates at 15 min

The pathological results are shown in Table 2. Other malignancies in the conventional LLLS group included one metastatic lung cancer, one metastatic renal cell cancer, one cholangiocellular carcinoma, and one metastatic gastrointestinal stromal tumor. Another malignancy in the RPLLLS group was one cholangiocellular carcinoma. Benign tumors in the conventional LLLS group included one hemangioma, one hepatocellular adenoma, and two infectious cysts. There were no significant differences between the two groups in terms of the largest tumor diameter (30.0 mm vs. 27.0 mm, *p* = 0.552), patients with multiple lesions (18.9% vs. 8.3%, *p* = 0.388), length of the surgical margin (12.0 mm vs. 19.0 mm, *p* = 0.278), and positive surgical margin rate (2.7% vs. 0%, *p* = 0.565).

**Table 2:** Pathologic results

Characteristics	Conventional LLLS *n* = 37	RPLLLS *n* = 12	*p* value
Diagnosis			0.245
HCC	13 (35.1)	2 (16.7)	
CRLM	16 (43.3)	9 (75.0)	
Other malignancy	4 (10.8)	1 (8.3)	
Benign	4 (10.8)	0	
Largest tumor diameter (mm)	30.0 (10.0–160.0)	27.0 (6.0–65.0)	0.552
Multiple tumors	7 (18.9)	1 (8.3)	0.388
Surgical margin (mm)	12.0 (0–40.0)	19.0 (5.0–60.0)	0.278
Positive surgical margin	1 (2.7)	0	0.565

*HCC* hepatocellular carcinoma, *CRLM* colorectal liver metastases

As shown in Table 3, operative and postoperative outcomes were compared between the two groups. Junior surgeons performed 62.2% of the conventional LLLSs (23 out of 37 patients) and 58.4% of the standardized RPLLLSs (7 out of 12 patients). All patients underwent successful LLLS, and no patient required conversion to laparotomy. The Pringle maneuver was not used in any patient. There were no significant differences between the two groups in operative time (121.0 min vs. 113.5 min, *p* = 0.387), blood loss (13.0 mL vs. 8.5 mL, *p* = 0.518), length of hospital stay (7.0 days vs. 7.0 days, *p* = 0.408), and morbidity rate (2.7% vs. 0%, *p* = 0.565). None of the patients required a blood transfusion (not shown). As for the postoperative complications, one patient in the conventional LLLS group required abdominal drainage for minor leakage of bowel contents due to intestinal injury following adhesiolysis. No mortality was reported among all of the patients.

**Table 3:** Surgical outcomes

Outcomes	Conventional LLLS *n* = 37	RPLLLS *n* = 12	*p* value
Operated by junior surgeon	23 (62.2)	7 (58.4)	0.813
Operative time (min)	121.0 (68.0–269.0)	113.5 (65.0–200.0)	0.387
Blood loss (mL)	13.0 (1–119)	8.5 (1–162)	0.518
Conversion to laparotomy	0	0	NA
Length of hospital stay (days)	7.0 (4.0–65.0)	7.0 (4.0–10.0)	0.408
Morbidity (Clavien–Dindo ≥ II)	1 (2.7)	0	0.565
Mortality	0	0	NA

*NA* not applicable

## Discussion

LLLS is one of the standard procedures in LLR and represents a safe and effective approach in the hands of trained surgeons with experience in hepatobiliary and laparoscopic surgery [1]. However, there are still few reports on conventional LLLS, single-port LLLS, or RPLLLS performed by junior surgeons. Several reports have shown that there are no significant differences between single-port LLLS and conventional LLLS groups in operative time, blood loss, length of hospital stay, and incidences of postoperative complications [11, 21]. Moreover, recent reports indicate that single-port LLLS exhibits comparable effectiveness and safety compared to conventional LLLS and can effectively reduce postoperative pain and improve cosmetic outcomes [22]. However, single-port LLLS is more complex for junior surgeons because it involves instrumental crowding and clashing. Furthermore, the lack of training programs focused on this topic and its gentle learning curve make LLR technically difficult [23].

This study shows that our standardized RPLLLS technique is safe and as feasible as conventional LLLS. Moreover, the standardized RPLLLS technique’s surgical results, such as blood loss and morbidity rate, are not inferior; rather, they are preferable to those cited in previous reports on single-port LLLS [11, 21, 24, 25]. However, operative time was relatively long compared to some single-incision LLLS studies [21, 22, 26, 27]. Two factors may explain the relatively long operation times in the present study. First, most of the LLLSs and RPLLLSs were performed by junior surgeons. The median operative time in the LLLS group was 105 min (minimum 68 min and max 170 min) for experienced surgeons and 130 min (minimum 84 min and max 269 min) for junior surgeons. The median operative time in the RPLLLS group was 105 min (minimum 93 min and max 115 min) for experienced surgeons and 121 min (minimum 65 min and max 200 min) for junior surgeons. Second, the percentage of a history of previous abdominal surgery was high: 56.8% for the conventional LLLS group and 91.7% for the RPLLLS group. Only one study reported the percentage of a history of previous abdominal surgery, and it was significantly lower: 12.8% for conventional LLLS and 18.2% for single-port LLLS [27]. Other studies did not report the percentage of a history of previous abdominal surgery [21, 22, 26]. When focusing on operative time, these points need to be considered.

The surgical indications of RPLLLS are generally identical to those of conventional LLLS. The placement of one epigastric port offers the following merits: (1) safe access to the left coronary and triangular ligaments; (2) safe access to the left hepatic vein; (3) easy assistance in liver transection procedures without instrumental crowding; and (4) capability to be performed in the same manner as conventional LLLS. In emergent situations, operations such as compression through the epigastric port enable the control of bleeding before converting to a formal open approach, as the already existing 40-mm incision make it easy to approach the bleeding point promptly. Notably, to prevent critical complications, the surgeon must be prepared to grab the central side of the left hepatic vein through the epigastric port to assuage bleeding if the stapling device is misfired (Fig. 2F). As for the other standard techniques utilized in this standardized RPLLLS, our previous report on the standardized LLLS demonstrates that using an Endoloop™ to tract the round ligament and the linear staplers to dissect the portal pedicles and left hepatic vein is effective for a safe and feasible operation [19]. Additionally, surgical ports with a large outer cap, such as GelPOINT™, help increase the instrument distance. The use of flexible endoscopes may also improve visualization of the surgical field [23]. The transection line in LLLS and the umbilical incision must be located on the same axis as the vector of the instrument work [11, 22, 28]. The characteristics of this LLLS help prevent the crossing over of the transection or hemostatic device and the endoscope.

Although LLLS is a feasible and safe procedure that is associated with a quick, smooth learning curve, the number of such procedures made available to junior surgeons is relatively limited. LLLS techniques, including mobilization of the liver, transection and hemostasis of the parenchyma, and dissection of the portal pedicles or left hepatic vein using an endoscopic linear stapler, provide junior surgeons with considerable experiences. The standardized RPLLLS also has advantage over conventional one in terms of feasibility and safety, in addition to being an ideal training procedure. This procedure may be beneficial, not only for junior surgeons but also for surgeons considering an introduction to the performance of more complex laparoscopic hepatectomies, such as single-port LLR and laparoscopic left lobectomy.

This study had certain limitations. First, we were unable to perform an effective statistical analysis due to the small sample size. A study with a larger sample size, such as a high-quality randomized controlled trial, is needed to confirm the conclusions. Second, between 2013 and 2017, five patients underwent the standardized RPLLLS conducted by expert surgeons. Throughout this period, the conventional LLLS or the standardized RPLLLS was performed according to the operator’s preference. Since 2018, the standardized RPLLLS has been selected for all applicable patients. Third, because the data were collected retrospectively, postoperative pain scores and cosmetic results, which might have revealed additional benefits of RPLLLS, were unavailable in the present study. Fourth, this study did not show any differences in hospital stays and had relatively long hospital stays (median 7 days), which may be explained by Japan’s national health insurance. Although the lengths of hospital stays are mainly determined by physicians’ clinical judgment, patients and their family members often participate in determining discharge dates. It may be difficult to compare the lengths of hospital stays in Japan with those in other countries. Finally, long-term outcomes were not evaluated in the present study; however, follow-ups with patients will continue to be carried out.

In conclusion, our RPLLLS procedure has been standardized, is reproducible, and is as safe and feasible as conventional LLLS. It is also preferable to single-port LLLS. Therefore, this standardized RPLLLS is a feasible and safe alternative to conventional LLLS and may become the ideal training procedure for both junior surgeons and surgeons aiming to learn to more complex procedures.

## Data Availability

All data generated or analyzed during this study are included in this published article.

## References

[1] Buell JF, Cherqui D, Geller DA, O’Rourke N, Iannitti D, Dagher I, Koffron AJ, Thomas M, Gayet B, Han HS, Wakabayashi G, Belli G, Kaneko H, Ker CG, Scatton O, Laurent A, Abdalla EK, Chaudhury P, Dutson E, Gamblin C, D’Angelica M, Nagorney D, Testa G, Labow D, Manas D, Poon RT, Nelson H, Martin R, Clary B, Pinson WC, Martinie J, Vauthey JN, Goldstein R, Roayaie S, Barlet D, Espat J, Abecassis M, Rees M, Fong Y, McMasters KM, Broelsch C, Busuttil R, Belghiti J, Strasberg S, Chari RS, World Consensus Conference on Laparoscopic Surgery (2009). The international position on laparoscopic liver surgery: The Louisville Statement, 2008. Ann Surg.

[2] Nguyen KT, Marsh JW, Tsung A, Steel JJ, Gamblin TC, Geller DA (2011). Comparative benefits of laparoscopic vs open hepatic resection: a critical appraisal. Arch Surg.

[3] Nguyen KT, Gamblin TC, Geller DA (2009). World review of laparoscopic liver resection-2,804 patients. Ann Surg.

[4] Azagra JS, Goergen M, Gilbart E, Jacobs D (1996). Laparoscopic anatomical (hepatic) left lateral segmentectomy-technical aspects. Surg Endosc.

[5] Kaneko H, Takagi S, Shiba T (1996). Laparoscopic partial hepatectomy and left lateral segmentectomy: technique and results of a clinical series. Surgery.

[6] Azagra JS, Goergen M, Brondello S, Calmes MO, Philippe P, Schmitz B (2009). Laparoscopic liver sectionectomy 2 and 3 (LLS 2 and 3): towards the “gold standard”. J Hepatobiliary Pancreat Surg.

[7] Benzing C, Krenzien F, Atanasov G, Seehofer D, Sucher R, Zorron R, Pratschke J, Schmelzle M (2015). Single incision laparoscopic liver resection (SILL)—a systematic review. GMS Interdiscip Plast Reconstr Surg DGPW.

[8] Abd Ellatif ME, Askar WA, Abbas AE, Noaman N, Negm A, El-Morsy G, El Nakeeb A, Magdy A, Amin M (2013). Quality-of-life measures after single-access versus conventional laparoscopic cholecystectomy: a prospective randomized study. Surg Endosc.

[9] Lurje G, Raptis DA, Steinemann DC, Amygdalos I, Kambakamba P, Petrowsky H, Lesurtel M, Zehnder A, Wyss R, Clavien PA, Breitenstein S (2015). Cosmesis and body image in patients undergoing single-port versus conventional laparoscopic cholecystectomy: a multicenter double-blinded randomized controlled trial (SPOCC-trial). Ann Surg.

[10] Aikawa M, Miyazawa M, Okamoto K, Toshimitsu Y, Okada K, Ueno Y, Yamaguchi S, Koyama I (2012). Single-port laparoscopic hepatectomy: technique, safety, and feasibility in a clinical case series. Surg Endosc.

[11] Aldrighetti L, Ratti F, Catena M, Pulitano C, Ferla F, Cipriani F, Ferla G (2012). Laparoendoscopic single site (LESS) surgery for left-lateral hepatic sectionectomy as an alternative to traditional laparoscopy: case-matched analysis from a single center. Surg Endosc.

[12] Shetty GS, You YK, Choi HJ, Na GH, Hong TH, Kim DG (2012). Extending the limitations of liver surgery: outcomes of initial human experience in a high-volume center performing single-port laparoscopic liver resection for hepatocellular carcinoma. Surg Endosc.

[13] Tayar C, Subar D, Salloum C, Malek A, Laurent A, Azoulay D (2014). Single incision laparoscopic hepatectomy: advances in laparoscopic liver surgery. J Minim Access Surg.

[14] Berardi G, Igarashi K, Wakabayashi G (2019). Laparoscopic liver resection-education and training. Transl Gastroenterol Hepatol.

[15] Guilbaud T, Birnbaum DJ, Berdah S, Farges O, Beyer Berjot L (2019). Learning curve in laparoscopic liver resection, educational value of simulation and training programmes: a systematic review. World J Surg.

[16] Hasegawa Y, Nitta H, Takahara T, Katagiri H, Baba S, Takeda D, Makabe K, Wakabayashi G, Sasaki A (2017). Safely extending the indications of laparoscopic liver resection: when should we start laparoscopic major hepatectomy?. Surg Endosc.

[17] Abu Hilal M, Pearce NW (2008). Laparoscopic left lateral liver sectionectomy: a safe, efficient, reproducible technique. Dig Surg.

[18] Cai X, Li Z, Zhang Y, Yu H, Liang X, Jin R, Luo F (2014). Laparoscopic liver resection and the learning curve: a 14-year, single-center experience. Surg Endosc.

[19] Hasegawa Y, Nitta H, Sasaki A, Takahara T, Ito N, Fujita T, Kanno S, Nishizuka S, Wakabayashi G (2013). Laparoscopic left lateral sectionectomy as a training procedure for surgeons learning laparoscopic hepatectomy. J Hepatobiliary Pancreat Sci.

[20] Linden BC, Humar A, Sielaff TD (2003). Laparoscopic stapled left lateral segment liver resection–technique and results. J Gastrointest Surg.

[21] Hu M, Zhao G, Wang F, Xu D, Liu R (2014). Single-port and multi-port laparoscopic left lateral liver sectionectomy for treating benign liver diseases: a prospective, randomized, controlled study. World J Surg.

[22] Cheng Y, Jiang ZS, Xu XP, Huang WF, He GL, Zhou CJ, Qin JS, Gao Y, Pan MX (2019). Single-port laparoscopic surgery is feasible and safe for hepatic left lateral sectionectomy for benign liver lesions. Gastroenterol Res Pract.

[23] Gkegkes ID, Iavazzo C (2014). Single incision laparoscopic hepatectomy: a systematic review. J Minim Access Surg.

[24] Struecker B, Haber P, Ollinger R, Bahra M, Pascher A, Pratschke J, Schmelzle M (2018). Comparison of single-port versus standard multiport left lateral liver sectionectomy. Surg Innov.

[25] Wang X, Li X, Cheng H, Zhang B, Zhong H, Wang R, Zhong B, Cao Q (2019). Single-port inflatable mediastinoscopy combined with laparoscopic-assisted small incision surgery for radical esophagectomy is an effective and safe treatment for esophageal cancer. J Gastrointest Surg.

[26] Tsai KY, Chen HA, Wang WY, Huang MT (2020). Long-term and short-term surgical outcomes of single-incision laparoscopic hepatectomy on anterolateral liver segments. Surg Endosc.

[27] Wang JC, Pan Y, Chen J, Hu D, Tuoheti Y, Zhou Z, Xu L, Chen J, Chen M, Zhang Y (2020). Single versus multiple port laparoscopic left lateral sectionectomy for hepatocellular carcinoma: a retrospective comparative study. Int J Surg.

[28] Ban D, Kudo A, Irie T, Ochiai T, Aihara A, Matsumura S, Tanaka S, Tanabe M (2015). Advances in reduced port laparoscopic liver resection. Asian J Endosc Surg.

